# Does lake eutrophication support biological invasions in rivers? A study on *Dreissena polymorpha* (Bivalvia) in lake–river ecotones

**DOI:** 10.1002/ece3.8013

**Published:** 2021-08-13

**Authors:** Robert Czerniawski, Tomasz Krepski

**Affiliations:** ^1^ Department of Hydrobiology Institute of Biology University of Szczecin Szczecin Poland

**Keywords:** Carlson Index, Dreissenidae, fluvial‐lacustrine ecotones, invasive species, zebra mussel

## Abstract

The zebra mussel (*Dreissena polymorpha*) has all traits required to effectively colonize the aquatic environment and consequently reduce the diversity of native bivalves. We hypothesized that the zebra mussel chooses lake outlets characterized by medium current velocity and good food conditions. Here, we analyzed differences between bivalve abundances in lake outlets with varying environmental conditions such as the Carlson Index (trophy status), depth, width, current velocity, bed vegetation coverage, and type of bottom substrate. The results showed that the zebra mussel inhabits outlets that provide food (high trophy outlets) and have a mineral bed and a medium current velocity (ca. 0.2–0.3 m/s). The following main factors seem to be favorable for colonizing such outlets: (1) easy access to high amounts of food due to the increased density of the suspension drifting from the lake and (2) easy transport of the zebra mussel larvae from the lake to the downstream. The zebra mussel larvae drifting with the current may colonize the downstream. An increase in lake trophy may indirectly cause an increase in biological invasions in rivers.

## Graphical abstract

Zebra mussel is worldwide invasive species which ecology in the river–lake ecotone is unknown. Dreissenids were more numerous in the outlets from highly eutrophicated lakes, and the mineral bottom and high water current velocity support the colonization. Lake pollution may positively affect the zebra mussel colonization in rivers.

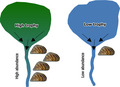

## 1 INTRODUCTION

Invasive species of bivalves have emerged in waters across Europe and America in recent decades (Benson, 2014; Karatayev et al., 2007; Łabęcka & Domagala, 2018; Mills et al., 1996; Sousa et al., 2014). Among these bivalve species, the zebra mussel (*Dreissena polymorpha*) is considered to be the most invasive and expansive species (Kobak & Ryńska, 2014; Sousa et al., 2011, 2014). The zebra mussel has all traits required to effectively colonize the environment and consequently reduce the diversity of native bivalves. These traits include short life cycle, free‐living larval forms that float and drift, high tolerance to changes in environmental conditions, high feeding efficiency, and the ability to create multilayer colonies in small bottom areas and to grow on other mussels (Dzierżyńska‐Białończyk et al., 2018; Karatayev et al., 2007; Mills et al., 1996; Sousa et al., 2014). The zebra mussel very effectively filters suspended solids, thus considerably improving water clarity (Collas et al., 2020; Mills et al., 1993; Pace et al., 1998). However, the species excretes high amounts of phosphorus into water (Orlova et al., 2004; Sousa et al., 2014; Wojtal‐Frankiewicz & Frankiewicz, 2011; Wojtal‐Frankiewicz et al., 2010). Hence, the presence of the zebra mussel leads to a significant increase in dissolved inorganic phosphorus in freshwater areas and a secondary increase in trophic status (Orlova et al., 2004). Accessibility to numerous ecological niches and potential habitats, not populated by competitive species, facilitates colonization opportunities for the zebra mussel. Potential habitats of the zebra mussel include lake–river ecotones, where this species is observed in amounts that vary with environmental conditions. For past several years, researchers have attempted to elucidate the reasons for colonization of new areas by invasive dreissenids. Although several studies have explored possibilities for monitoring, limiting the presence of alien dreissenids, or eliminating them altogether, these issues remain unresolved (Martin et al., 1993; Mehler et al., 2018; Mills et al., 1993; Molloy et al., 2013; Ricciardi et al., 1995; Simberloff, 2014). Furthermore, these issues are becoming more severe because invasive species such as the zebra mussel keep emerging in new areas and effectively replacing native species.

Lotic waters encourage passive drifting and spread of the zebra mussel larvae. However, such waters frequently supply less food than stagnant waters, which are the preferred habitat of the zebra mussel. Hence, a lake–river ecotone zone is a place that meets both criteria for a good habitat for this species. In small lake–river ecotones present in lake outlets, trophic conditions and physicochemical characteristics are the same or similar to those of lake waters, whereas the hydrological conditions are the same as those in streams or small rivers (Czerniawski & Domagała, 2013; Hieber et al., 2002; Wotton, 1995). Massive concentration of food (suspended solids) in the narrow bed of the outlet and easy access to food drifting from the lake make lake–river ecotones a good habitat for bivalves such as the zebra mussel or other filter feeders (Czerniawski et al., 2016; Richardson & Mackay, 1991). Furthermore, good nutritional conditions of lakes (high trophy status) may contribute to a high abundance of the zebra mussel in the outlets because invasive and expansive species such as the zebra mussel can tolerate significant changes in the trophic status (Bates et al., 2013; Leuven et al., 2009; Ricciardi et al., 1995; Zerebecki & Sorte, 2011). This further contributes to an increase in the concentration of the zebra mussel in downstreams of the outlets. Free‐flowing veligers of the zebra mussel, which are components of zooplankton community, drift downstream, and if they encounter good conditions, they colonize the downstream, thus reducing biodiversity and changing the status of trophic conditions. Given the constantly progressing anthropogenic eutrophication of lakes, this serious issue needs to be addressed.

Few studies have investigated the dispersal of larval and adult zebra mussels in streams or lake outlets. Horvath and Lamberti (1997) considered that the upstream lake provides a mechanism such as drifting macrophytes by which the attached adult zebra mussels can invade outlet streams. Horvath et al. (1999) concluded that zebra mussel communities cause an increase in the abundance of macroinvertebrates in lake outlets, which is probably related to the increased complexity of hard substrata provided by zebra mussels. Gray (2005) reported that small lake outlet streams contribute significant numbers of the dispersed veligers of zebra mussels to larger rivers. Horvath et al. (1996), Miller and Haynes (1997), and Horvath and Lamberti (1999) examined the role of streams in the spatial dispersion of zebra mussels in running water systems. None of these studies, however, examined the influence of environmental conditions on the colonization of lake outlets by zebra mussels and did not determine the influence of trophic conditions of lakes on zebra mussel occurrence in their outlets, which is important because of the constant and increasing threat to lakes from humans.

The primary goal of any species is to maintain its population. The species must find a habitat with constant access to food and conditions that allow them to reproduce, develop, and colonize the area. We hypothesized that the zebra mussel chooses lake outlets characterized by relatively high current velocity and good trophic conditions and prefers to inhabit outlets of highly eutrophicated lakes. We believe that studying the presence of other bivalves in the outlets would not provide insightful findings. Thus, we considered the Sphaeriidae and Unionidae species as a reference point for the presence and abundance of the zebra mussel. By knowing what structures the zebra mussel forms in lake outlets and what factors shape those structures, we can better understand how to control the spread of this invasive species in rivers.

## 2 MATERIALS AND METHODS

**FIGURE 1:**
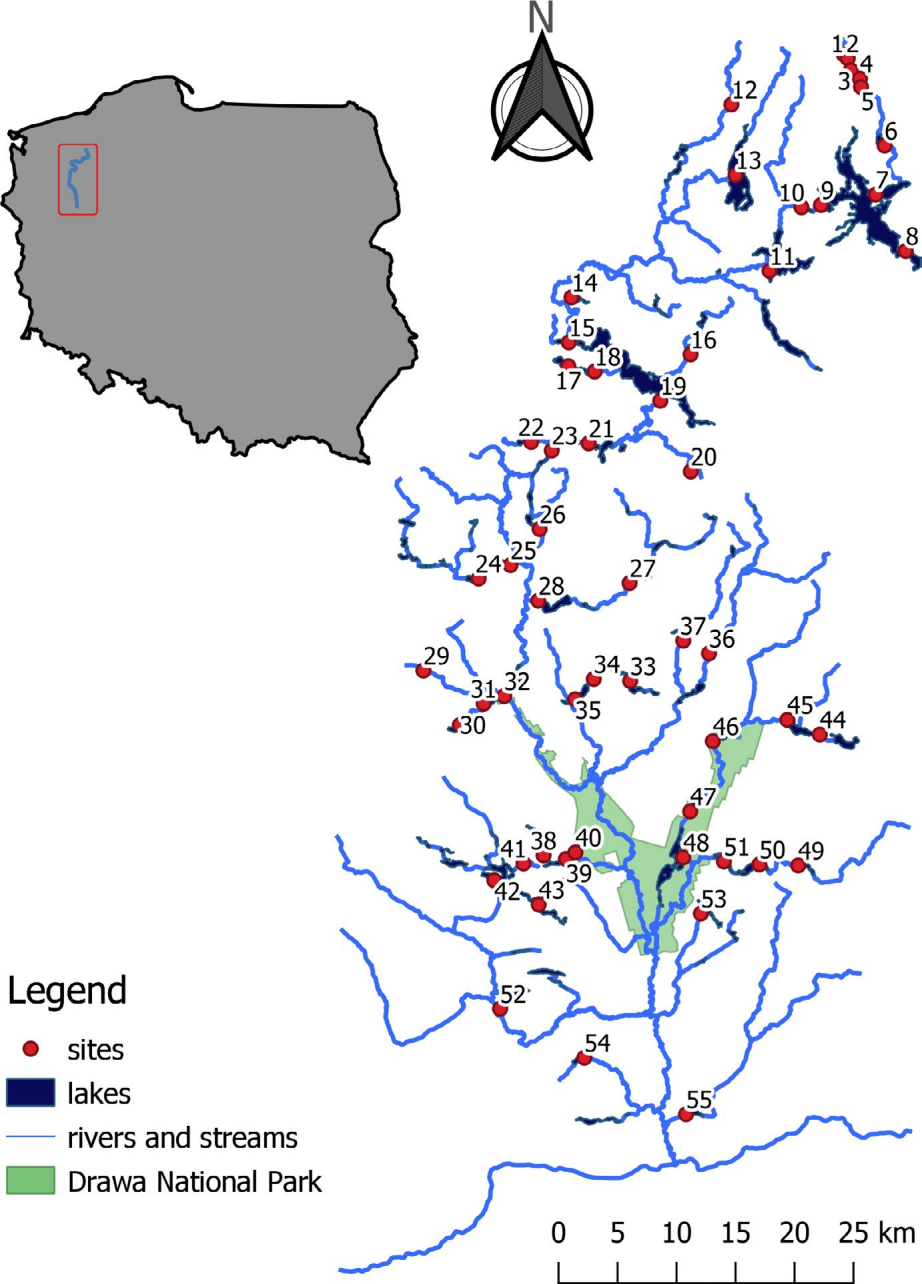
Map of the sampling sites in Drawa River catchment

The study was performed in the catchment area of the Drawa River (GPS: 53°20ʹ25ʺ N; 15°46ʹ30ʺ E – the middle Drawa), which is a 190‐km‐long quaternary tributary of the Odra River (Figure 1). The Drawa River is situated in the Pomeranian Lake District, NW, Poland. Springs of the Drawa River lie at an altitude of 150 m a. s. l. The mean slope of the riverbed is 0.59 m/km, and the catchment area of the Drawa River is 3,198 km^2^. Samples of bivalves were collected from 55 lake outlets in July 2016. An area of approximately 50 m^2^ was selected for the study, starting from the shoreline of the lake downstream. Samples of macrozoobenthos were collected at the point where the water current changed from the cumulative to the rectilinear. The samples were collected using a Surber Sampler (sampling surface 0.0625 m^2^) by disturbing the bottom at four places: Two places were located at the middle of the outlet and one at each shore. The abundance of the organisms was counted per square meter. Subsamples were put together and treated as one sample. We decided to use a Surber sampler because of the small size of Sphaeriidae clams. To avoid “double zeros,” an entire 50‐m section of the outlet was checked for the absence of zebra mussel.

We analyzed differences in bivalve abundances in lake outlets with varying environmental conditions such as the Carlson Index (trophy status), depth, width, current velocity, bed vegetation coverage, and type of bottom substrate (Table S1). We divided the outlet sections according to the value ranges of the above parameters, as shown in Table 1.

**TABLE 1:** Mean values ±*SD* of abundance of bivalves in outlets characterized by: Trophy—high, medium, and low values of Carlson Index; Depth—low, medium, and high values of depth; Width—very small, small, medium, large, and very large values of bed width; Current—very low, low, medium, and high values of current velocity; Vegetation coverage—low medium, high, and very high values of percentage vegetation coverage; Bottom—different bottom type (in parentheses are frequency percentage values at sites)

	*n*	Dreissenidae	Sphaeriidae	Unionidae
Trophy				
High trophy (55–65)	27	363 ± 1,020 a	188 ± 259 a	7 ± 18 a
Medium trophy (46–54)	21	17 ± 50 a	421 ± 692 a	4 ± 9 a
Low trophy (37–44)	7	–	48 ± 37 a	–
Depth				
Low (0.1–0.15 m)	19	2 ± 9 b	196 ± 300 b	1 ± 1 b
Medium (0.2–0.3 m)	25	153 ± 676 bc	390 ± 641 b	8 ± 20 b
High (0.4–1.2 m)	11	573 ± 1,210 c	69 ± 51 b	7 ± 9 b
Width				
Very small (0.15–0.70 m)	12	–	215 ± 332 d	1 ± 1 d
Small (1.0–1.6 m)	13	6 ± 15 d	466 ± 650 d	1 ± 4 d
Medium (2.5–5 m)	15	252 ± 858 d	315 ± 566 d	12 ± 24 d
Large (6–10 m)	9	673 ± 1,318 d	43 ± 40 d	5 ± 10 d
Very large (11–22 m)	6	43 ± 55 d	82 ± 54 d	4 ± 5 d
Current				
Very low (<0.08 m/s)	19	2 ± 9 f	264 ± 364 f	1 ± 1 f
Low (0.1–0.2 m/s)	16	1 ± 2 f	204 ± 200 f	4 ± 7 f
Medium (0.22–0.39 m/s)	17	595 ± 1,230 g	343 ± 745 f	11 ± 23 f
High (0.5–0.6 m/s)	3	–	48 ± 68 f	3 ± 4 f
Vegetation coverage				
Low (0%–20%)	29	226 ± 794 hr	340 ± 611 hr	2 ± 4 hr
Medium (30%–40%)	6	19 ± 19 hr	96 ± 103 hr	6 ± 12 hr
High (50%–60%)	9	389 ± 1,084 hr	176 ± 177 hr	18 ± 30 hr
Very high (70%–100%)	11	–	203 ± 339 hr	–
Bottom				
POM/sand	30	123 ± 631 i (10)	291 ± 499 i (93)	6 ± 19 i (23)
Sand/stones	3	61 ± 86 ij (100)	74 ± 76 i (100)	3 ± 5 i (67)
Sand/gravel	12	520 ± 1,224 j (75)	73 ± 104 i (100)	5 ± 9 i (41)
Sand	10	5 ± 13 ij (22)	442 ± 719 i (84)	2 ± 5 i (10)

Note

Different letters in column indicate significant differences between abundance of individuals (*p* < .05).

At each site, we determined water velocity, width, and depth with an electromagnetic water flow sensor (OTT Hydromet, Germany). We also visually estimated total vegetation cover of bed for each one‐meter stream‐long plot (across the width of the stream). Then, we estimated the average percentage coverage for 50‐m outlet section. We also examined a 50‐m transect at each site for macrophytes. We measured water transparency with a Secchi disk in each lake from which the watercourses flowed. Lake trophic status was expressed in terms of the Carlson Index, TSI_SD_ = 10 (6–log_2_ *SD*), where *SD* is the maximum depth in meters at which the Secchi disk was visible. The higher the Carlson Index, the higher is the trophy status of a lake. The environmental variables and their ranges are shown in Table S1. We visually determined the total percentage of the dominant bed substrate from a Surber sampler square, before mollusks were sampled (particulate organic matter [POM], sand, gravel, stones, unified result). We specified the substrate mix if the bed was covered rather evenly by two substrates.

One‐way Kruskal–Wallis test (*p* < .05) was used to verify significant differences between bivalve abundances in lake outlets with varying conditions such as the Carlson Index (trophy status), depth, width, current velocity, bed vegetation coverage, and type of bottom substrate. To determine significant differences in the abundances of bivalves between different lake outlet sections, we performed post hoc multiple comparisons of mean ranks for all groups with Bonferroni correction (*p* < .05). Cluster analysis based on Euclidean distance was performed to identify groups of outlets with similar bottom substrate types in the context of bivalve abundance. Principal components analysis (PCA) was used to show similarity between outlets with regard to the absence and presence of zebra mussels in environmental conditions such as the Carlson Index (trophy status), depth, width, current velocity, and bed vegetation coverage (used together). The PCAs were performed using package “stats” in R software (R Core Team, 2020). We used *t* test (*p* < .05) to verify significant differences between outlets with regard to the absence and presence of zebra mussels in environmental conditions such as the Carlson Index (trophy status), depth, width, current velocity, and bed vegetation coverage (used separately). Spearman's correlation (*p* < .05) was used to determine the influence of independent environmental variables of the outlets on the abundance of bivalves and to define the most important variables affecting bivalve communities in lake outlets. Additionally, the canonical correspondence analysis (CCA) was used to determine the influence of environmental factors on the abundance of bivalves. The analyses were performed using Vegan 2.5–7 software (Oksanen et al., 2020).

We studied the Sphaeriidae and Unionidae species to verify whether these species respond to the same environmental conditions as the zebra mussel. The zebra mussel is the only mollusk out of the three discussed species that develops into free‐living larvae, and this consequently may affect the differences in the abundance of the studied bivalve groups in various lake outlets. To keep the transparency in the tables, for Dreissenidae, we used the family level instead of species name. Every individual of the Dreissenidae family belongs to *D. polymorpha*.

## 3 RESULTS

### 3.1 Bivalve abundance and frequency, and environmental conditions

The zebra mussel and the Sphaeriidae and Unionidae species were present in 31%, 100%, and 25% of the outlets, respectively (Table S1). Abundance of the zebra mussel and the Sphaeriidae and Unionidae species ranged from 0 to 3,884 ind/m^2^, 4 to 2,416 ind/m^2^, and 0 to 96 ind/m^2^, respectively.

The Carlson Index ranged from 37 to 65, indicating the lowest and highest trophy status of lakes (Table S1). Depth of the outlets ranged from 0.1 to 1.2 m and the width from 0.15 to 22 m. Current velocity ranged from 0.02 to 0.6 m/s. Vegetation covered from 0% to 100% of the bed in the outlet section. We observed 30 outlet sections with beds composed of POM–sand substrate, 3 with sand–stone substrate, 12 with sand–gravel substrate, and 10 with sand substrate.

**FIGURE 2:**
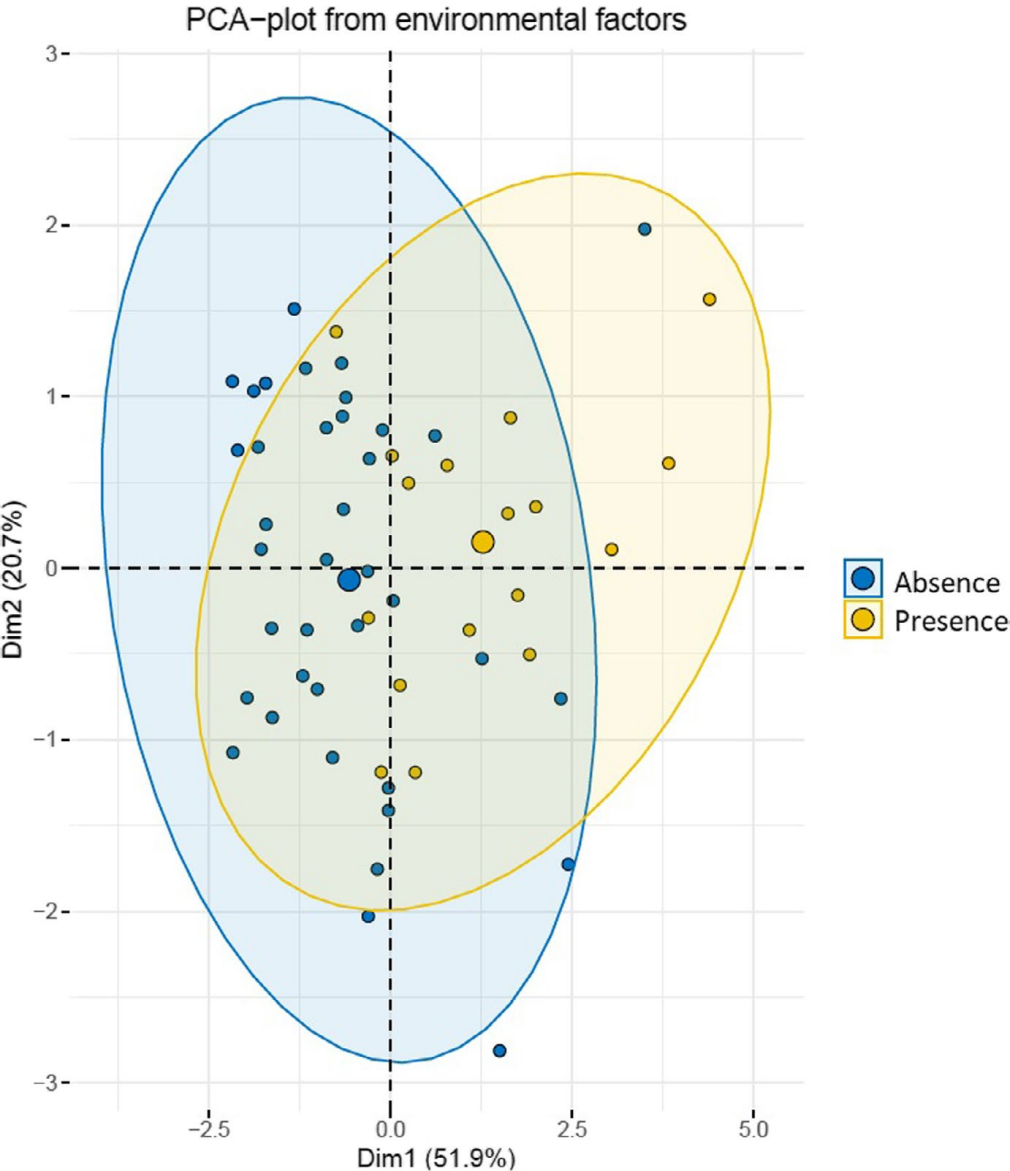
PCA grouping sites with the presence and absence of zebra mussel based on environmental conditions (without type of bed substrate)

PCA based on all environmental conditions together (without type of bottom substrate) showed differences between sites with and without the presence of zebra mussel (Figure 2). More clearer differences showed results of *t* test (Table 2). Sites with the presence of zebra mussel showed significantly higher value of the Carlson Index and all hydrological factors and significantly lower amount of surface covered by macrophytes than sites without zebra mussel (*p* < .05) (Table 2).

**TABLE 2:** Mean values of environmental conditions and result of *t* test about significant differences in environmental condition values of outlets between sites with the absence and presence of zebra mussel (*p* < .05)

Environmental conditions	Sites with the absence of zebra mussel *n* = 38	Sites with the presence of zebra mussel *n* = 17	*t*	*p*
Carlson Index	52.0 ± 7.6	57.3 ± 5.9	−2.5	.014
Depth (m)	0.2 ± 0.2	0.4 ± 0.3	−3.7	.000
Width (m)	3.2 ± 4.2	7.5 ± 6.4	−2.9	.005
Current velocity (m/s)	0.1 ± 0.1	0.3 ± 0.1	−3.7	<.001
Discharge (m^3^/s)	0.3 ± 0.6	1.3 ± 1.9	−3.1	.003
Vegetation coverage (%)	40.1 ± 32.6	17.1 ± 18.5	2.7	.009

### 3.2 Abundance of bivalves in various lake outlets

There were no significant differences in the abundances of bivalve groups between lakes with different trophies (the Carlson Index values). However, zebra mussel and the Unionidae were absent in the outlets of low trophy lakes. The density of zebra mussel was considerably higher in the outlets of high trophy lakes than in the medium one (Table 1).

The abundance of the zebra mussel was significantly lower in shallow outlets than in profound ones (*p* < .05) (Table 1). A similar but nonsignificant difference was observed between shallow outlets and those of medium depth (*p* > .05). No significant differences in the abundances of other bivalves were observed between outlets with different depths (*p* > .05).

According to bed width in the outlet, no significant differences were observed between various sites (*p* > .05). However, the abundance of the zebra mussel differed clearly between sites with small and large width of beds (*p* < .05) (Table 1). The zebra mussel was absent in outlets with a bed width <0.7 m.

Moreover, the abundances of the zebra mussel differed significantly among outlets with different current velocities (Table 1). In outlets with current velocity ranging from 0.22 to 0.39 m/s (considered by the authors as “average”), the zebra mussel achieved significantly higher abundances than that in outlets with low and exceptionally low current velocity (*p* < .05). Moreover, the zebra mussel was absent in 3 outlets with current velocity higher than 0.5 m/s.

According to the percentage of vegetation coverage in the outlets, no significant differences were observed in the abundances of any bivalves (*p* > .05) (Table 1). However, the zebra mussel and the Unionidae species were absent from samples collected in outlets with remarkably high vegetation coverage.

The zebra mussel achieved significantly higher abundances in outlets with a sand–gravel bed than in outlets with a POM–sand bed (*p* < .05) (Table 1). The abundances of the Sphaeriidae and Unionidae species did not differ across outlets with different types of bed substrate (*p* > .05).

**FIGURE 3:**
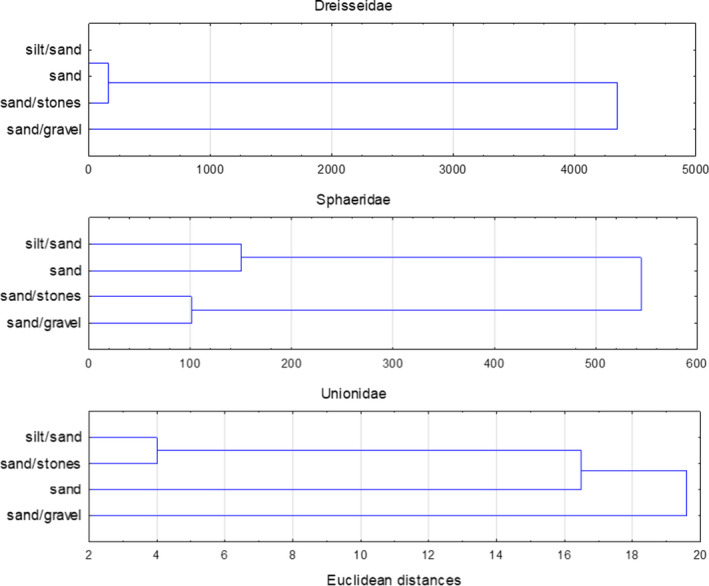
The dendrogram (cluster analysis) grouping bottom type of lake outlets based on the abundance of individuals of Dreissenidae, Sphaeriidae, and Unionidae

For the zebra mussel, cluster analysis showed similarities between sand and sand–stone beds (Figure 3). For the Sphaeriidae species, cluster analysis showed no clear similarities between the types of bed, but with regard to the abundance of the Unionidae species, most similarities were observed between POM–sand and sand–stone beds (Figure 3).

### 3.3 The relationship between abundance and environmental conditions

Spearman's correlation analysis showed that all hydrological conditions of the outlets were significantly positively correlated with the abundance of the zebra mussel (*p* < .05) (Table 3). Significant positive correlation was observed between the abundances of the zebra mussel and the Unionidae species (*r* = 0.28, *p* < .05).

**TABLE 3:** Significant Spearman correlations between abundance of bivalves (Dreissenidae, Sphaeriidae, Unionidae) (ind/m^2^) and environmental conditions of lake outlets

Environmental factors	Dreissenidae	Unionidae
Carlson Index	0.30	–
Depth	0.53	0.38
Width	0.45	0.33
Current velocity	0.48	–
Vegetation coverage	−0.29	–

**FIGURE 4:**
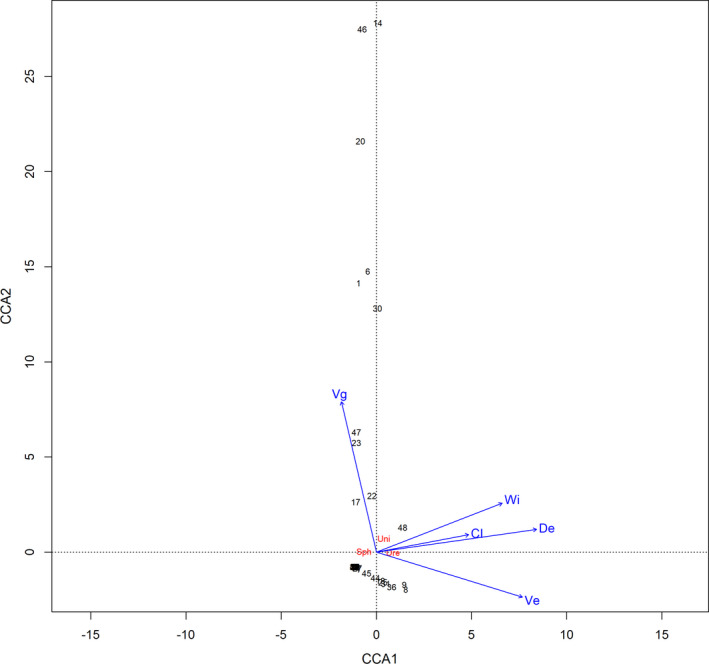
CCA ordination diagram of bivalve group abundance along with environmental factors: Dre, Dreissenidae; Uni, Unionidae; Sph, Sphaeriidae; Wi, width of the bed; De, depth of the outlet; Ve, current velocity; Vg, vegetation coverage; CI, Carlson Index. Eigenvalues: CCA1 = 0.5653, CCA2 = 0.0061

Both CCA axes explained 56% of the total variability in bivalve abundance (Figure 4). The first axis explained the majority of the variability. Zebra mussel abundance was positively associated with the depth and current velocity values. This relationship was also strong for the Carlson Index and bed width. All these variables correlated negatively with the abundance of the Sphaeriidae species. Zebra mussel abundance was negatively associated with the vegetation coverage. The Unionidae species were positively associated with similar variables (except for current velocity) such as the zebra mussel, but to a much smaller extent. The hydrological variables in particular justify remote distances between the three groups of bivalves.

## 4 DISCUSSION

### 4.1 Trophy status

The zebra mussel prefers to inhabit mesotrophic and moderately eutrophic lakes where it reaches higher densities than in eutrophic or highly eutrophicated lakes (Dorgelo, 1993; Mills et al., 1996; Stańczykowska & Lewandowski, 1992). This conclusion is not supported by our study results. Our study revealed that the zebra mussel occurred in higher abundance and at a higher number of sites in outlets of moderately eutrophicated or even highly eutrophicated lakes, that is, lakes with high or the highest values of the Carlson Index. This could be explained by the large amount of food for the zebra mussel, such as live organic matter, minor algae, and minor rotifers, present in these lakes (Wacker & Elert, 2003; Winkel & Davids, 1982; Wong & Levinton, 2005) or by the availability of the large amount of food that results in less competition and therefore higher number of mussels. Dorgelo (1993) observed bigger individuals of the zebra mussel in eutrophic lakes; however, their densities were higher in mesotrophic lakes. This shows that the zebra mussel achieves larger size of individuals (higher mass) in eutrophic lakes than in mesotrophic lakes; however, it cannot obtain higher densities in eutrophic lakes. A mineral substrate of bed and concentration of suspension in a small water volume in a narrow lake outlet make a good place to attach to hard substrate and rich food place that can be settled by filtrators in both eutrophic and mesotrophic lake outlets. In most cases, there is more food in lakes than in small rivers or streams, and this explains why the zebra mussel colonizes outlets where the entire water volume is rich in organic matter transported by the water drift (Pace et al., 1998).

### 4.2 Current velocity and depth

The zebra mussel inhabited outlets of eutrophicated lakes with a medium current velocity that flushes out organic matter from the lake into the outlet. Turbulently flowing water in lakes ensures a better food supply, which results in higher growth rates of zebra mussel individuals (Dorgelo, 1993). Studies on plankton drifting from lakes confirm that low current velocity and low turbulence limit the transport of organic matter from lakes to their outlets (Czerniawski & Domagała, 2013; Czerniawski & Pilecka‐Rapacz, 2011). Furthermore, the majority of zooplankton individuals resist current velocity lower than 0.1 m/s (Czerniawski & Sługocki, 2017). We also observed that the zebra mussel was not present in outlets with current velocity greater than 0.4 m/s. Therefore, too high current velocity hinders colonization of even highly eutrophicated outlets with a sand–gravel bed. On the one hand, high current velocity may prevent veligers from attaching to the bottom surface and cause the organic matter to drift more rapidly, thus hindering the zebra mussel's water filtration abilities. On the other hand, in our study, we found only 3 outlets with current velocity higher than 0.4 m/s, which is insufficient to explain whether current velocity higher than 0.4 m/s hinders filtration and attachment to the bottom surface. Large river habitats with coarse substrate and near‐bottom flow of 0.6–0.80 m/s or below 1.2 m/s are found to be most often occupied by the zebra mussel (Mehler et al., 2018; Sanz‐Ronda et al., 2014). However, habitats of large rivers are much different environmentally than those of small stream outlets of lakes, and therefore, comparing them with each other is not reasonable. We believe that this issue should be given appropriate attention and resolved in future studies. Invertebrates use water current to move downstream and colonize these areas (Allan & Castillo, 2007; Hayes et al., 2007; Lancaster et al., 1996). During such passive migration, organisms use water's gravitational energy and a bare minimum of their own energy to migrate long distances. Lake outlets also favor the passive transport of the zebra mussel larvae and their colonization of the downstream. Veligers spawned from lake populations are thought to be an important means of spread of zebra mussels into outflowing rivers (Horvath & Lamberti, 1997). This is possible due to the free‐living larval forms that may drift with the current from the outlet into the downstream and colonize favorable niches (Mills et al., 1996; Stańczykowska & Lewandowski, 1992). In contrast to the zebra mussel, larvae of other bivalves are not free‐living but are parasitic or develop in the parental embryo area in gills (Blazek & Gelnar, 2006; Taeubert et al., 2014). Because of its specific behavior, the zebra mussel inhabits lake outlets that provide food and migratory opportunities. Due to their free‐living nature and small size, the zebra mussel larvae may drift freely throughout the river volume like other planktonic organisms (Lazareva et al., 2016; Mills et al., 1995). Studies on zooplankton drift from lake outlets showed that zooplankton can drift passively up to several kilometers downstream (Basu & Pick, 1997; Pourriot et al., 1997). This ecotone is a perfect habitat for the invasive and expansive species such as the zebra mussel. However, high turbulence in streams can cause mortality of veligers and reduce the possibility of their dispersion (Horvath & Lamberti, 1999; Rehman et al., 2003). Naturally, an appropriate depth of the outlet section (>0.1 m) allows species survival, while current velocity also plays an essential role. Bowers and Szalay (2004) recorded low amount of the Unionidae species and very few colonies of the zebra mussel in shallow lake areas (10–35 cm), which indicates that water level fluctuations limited their distribution.

### 4.3 Types of bottom substrate

The zebra mussel cannot attach to the bottom of highly eutrophicated lakes because the bed is mainly organic and covered with POM (fine and coarse POM) (Dorgelo, 1993; Mills et al., 1996; Stańczykowska & Lewandowski, 1992). However, in the outlets of these lakes with an adequately fast current, the POM is flushed out from the bed, thus exposing mineral forms such as stones and gravel, to which the zebra mussel can attach itself (Bodamer & Ostrofsky, 2010; Mills et al., 1993; Patterson et al., 2005). In our present study, we identified only one site with a POM–sand bed where the amount of the zebra mussel was remarkably high (over 3,400 ind/m^2^). However, the zebra mussel may have attached to itself or to the remains of a weir that were present at the site. Additionally, the outlet was significantly covered with vegetation, to which the zebra mussel could have attached itself as well. This is, however, a more complex issue that is discussed in the next paragraph. We also expected that zebra mussel would prefer rocks and stones over the sand. In other studies, they were found on stones that were exposed out of the sand. However in our case, we do not observed that. Furthermore current velocities were too low to expose big stones and rocks. A positive and significant correlation between the abundances of the zebra mussel and the Unionidae species may suggest that the zebra mussel uses Unionidae as a hard substrate. However, we did not find any zebra mussel individuals attached to Unionidae.

### 4.4 Vegetation coverage

In the majority of the studied outlets, aquatic vegetation did not favor the colonization of the outlets by the zebra mussel. Dense vegetation may inhibit the current flow, thus limiting drifting of organic matter to the outlet. Secondly, in such cases, vegetation densely covering the bottom and plants growing from the bottom to the surface of the water may trap the drifting food (Mouton et al., 2019; Schulz et al., 2003). However, in one site, the abundance of bivalves was high, even though 50% of the bottom was covered by macrophytes. The only factor that differentiated this site from the other ones was the current velocity exceeding 0.2 m/s. The most likely explanation is that the aquatic vegetation did not inhibit the current flow nor limited the amount of organic matter drifting from the lake into the outlet. The zebra mussel sometimes attaches itself to aquatic plants; however, it does so when no hard substrate is available (Bodamer & Ostrofsky, 2010; Stańczykowska & Lewandowski, 1992).

Increased anthropogenic activity, habitat loss and degradation, introduction of invasive species, environmental pollution, diseases, and global climatic changes have led to an increase in the amount and density of invasive alien species (Cieplok & Spyra, 2020; Crooks et al., 2011; Jones & McDermott, 2018). This is a well‐known issue. By constant lake exploitation and pollution, humans contribute to an uncontrollable rapid growth of the trophy and an increase in the trophic status (Bertahas et al., 2006; Szyper & Gołdyn, 2002). The results of our present study demonstrate why human activity is unfavorable for lakes and may lead to an increase in the nutrient concentration and in the density of the zebra mussel in outlets and further downstream. High amounts of phosphorus excreted by the zebra mussel additionally boost the increase in eutrophication (Wojtal‐Frankiewicz & Frankiewicz, 2011; Wojtal‐Frankiewicz et al., 2010). Limiting the sources of inorganic and organic nutrients therefore seems to be necessary for several reasons. Firstly, it would inhibit the eutrophication processes, and secondly, it would reduce the colonization of outlets in high trophy lakes by alien and invasive dreissenids. Furthermore, the regulation and straightening of outlet beds increase the current velocity and decrease the ecological status (Bączyk et al., 2018; Stępień et al., 2019), which may also favor the colonization of outlets by the zebra mussel. In this case, the most optimal solution is to promote renaturalization of outlet beds and create meanders as well as riffle sequences that reduce or diversify current velocity (Palmer et al., 2010; Wohl et al., 2015).

The results of the present study suggest that the zebra mussel was absent at certain sites probably because it had not reached those areas yet. However, the fact that all the studied sites were located in close proximity and in one small catchment area rules out such an assumption. Thus, neither the lack of connections nor long distances limit the ability of the zebra mussel to spread to other sites. Moreover, the zebra mussel was observed in the Drawa catchment 40 years ago (Jasnowski & Jasnowska, 1982). Even in the areas where the zebra mussel was not present, we observed a few empty or pounded shells of this species. Therefore, the inability to disperse does not explain the absence of the zebra mussel from the outlets. It is more likely that their absence is caused by environmental conditions in the outlets, because all sites with bed having majority of POM in substrate and with high current velocity showed the absence of zebra mussels. Hence, to analyze the correlation between the number of mussels and environmental variables, we evaluated all sites together, including the presence of zebra mussels and without them.

## 5 CONCLUSION

The zebra mussel was observed more frequently and in greater amounts in bigger outlets and was absent in small outlets. The zebra mussel does not colonize small outlets with low current velocity. This species is more likely to colonize bigger outlets of highly eutrophicated lakes; however, it prefers outlets with medium current velocity (>0.2 m/s). Lake outlets are beneficial for the zebra mussel in two ways—firstly, by providing food sources, and secondly, by facilitating effective colonization of rivers. Therefore, it can be concluded that lake–river ecotones are hatcheries of the zebra mussel and play a strategic role in the colonization of the downstream sections of rivers. The results of the present study demonstrate that an increase in lake trophy may indirectly cause an increase in biological invasions in rivers.

## CONFLICT OF INTEREST

The authors have no conflict of interest to declare.

## AUTHOR CONTRIBUTION

**Robert Czerniawski:** Conceptualization (equal); Data curation (supporting); Formal analysis (equal); Investigation (equal); Methodology (equal); Supervision (lead); Writing‐original draft (lead). **Tomasz Krepski:** Conceptualization (equal); Data curation (lead); Formal analysis (equal); Investigation (equal); Methodology (equal); Visualization (lead); Writing‐review & editing (lead).

## Supporting information

Table S1

## Data Availability

The data used to generate the analysis presented in the paper are available in a Table S1. Data also were stored in Dryad (https://doi.org/10.5061/dryad.j3tx95xf2).

## References

[ece38013-bib-0001] Allan, J. D., & Castillo, M. M. (2007). Stream ecology: Structure and function of running waters. Springer Science & Business Media.

[ece38013-bib-0002] Bączyk, A., Wagner, M., Okruszko, T., & Grygoruk, M. (2018). Influence of technical maintenance measures on ecological status of agricultural lowland rivers – Systematic review and implications for river management. Science of the Total Environment, 627, 189–199. 10.1016/j.scitotenv.2018.01.235 29426140

[ece38013-bib-0003] Basu, B. K., & Pick, F. R. (1997). Phytoplankton and zooplankton development in a lowland, temperate river. Journal of Plankton Research, 19, 237–253. 10.1093/plankt/19.2.237

[ece38013-bib-0004] Bates, A. E., McKelvie, C. M., Sorte, C. J. B., Morley, S. A., Jones, N. A. R., Mondon, J. A., Bird, T. J., & Quinn, G. (2013). Geographical range, heat tolerance and invasion success in aquatic species. Proceedings of the Royal Society B‐Biological Sciences, 280, 20131958. 10.1098/rspb.2013.1958 PMC3813329 24266040

[ece38013-bib-0005] Benson, A. J. (2014). Chronological history of zebra and quagga mussels (Dreissenidae) in North America, 1988–2010. In T. F. Nalepa, & D. W. Schloesser (Eds.), Quagga and Zebra Mussels: Biology, Impacts, and Control, 2nd ed. (pp. 9–31). CRC Press.

[ece38013-bib-0006] Bertahas, I., Dimitriou, E., Karaouzas, I., Laschou, S., & Zacharias, I. (2006). Climate change and agricultural pollution effects on the trophic status of a Mediterranean lake. Acta Hydrochimica Et Hydrobiologica, 34, 349–359. 10.1002/aheh.200500637

[ece38013-bib-0007] Blazek, R., & Gelnar, M. (2006). Temporal and spatial distribution of glochidial larval stages of European unionid mussels (Mollusca: Unionidae) on host fishes. Folia Parasitologica, 53, 98–106. 10.14411/fp.2006.013 16898123

[ece38013-bib-0008] Bodamer, B. L., & Ostrofsky, M. L. (2010). The use of aquatic plants by populations of the zebra mussel (*Dreissena polymorpha*)(Bivalvia: Dreissenidae) in a small glacial lake. Nautilus., 124, 100–106.

[ece38013-bib-0009] Bowers, R., & Szalay, F. (2004). Effects of Hydrology on Unionids (Unionidae) and Zebra Mussels (Dreissenidae) in a Lake Erie Coastal Wetland. American Midland Naturalist, 151, 286–300.

[ece38013-bib-0010] Cieplok, A., & Spyra, A. (2020). The roles of spatial and environmental variables in the appearance of a globally invasive *Physa acuta* in water bodies created due to human activity. Science of the Total Environment, 744, 140928. 10.1016/j.scitotenv.2020.140928 32698048

[ece38013-bib-0011] Collas, F. P. L., Koopman, K. R., van der Velde, G., & Leuven, R. S. E. W. (2020). Quantifying the loss of filtration services following mass mortality of invasive dreissenid mussels. Ecological Engineering, 149, 105781. 10.1016/j.ecoleng.2020.105781

[ece38013-bib-0012] Crooks, J. A., Chang, A. L., & Ruiz, G. M. (2011). Aquatic pollution increases the relative success of invasive species. Biological Invasions, 13, 165–176. 10.1007/s10530-010-9799-3

[ece38013-bib-0013] Czerniawski, R., & Domagała, J. (2013). Reduction of zooplankton communities in small lake outlets in relation to abiotic and biotic factors. Oceanological and Hydrobiological Studies, 42, 123–131. 10.2478/s13545-013-0065-z

[ece38013-bib-0014] Czerniawski, R., & Pilecka‐Rapacz, M. (2011). Summer zooplankton in small rivers in relation to selected conditions. Open Life Sciences, 6, 659–674. 10.2478/s11535-011-0024-x

[ece38013-bib-0015] Czerniawski, R., & Sługocki, Ł. (2017). Analysis of zooplankton assemblages from man‐made ditches in relation to current velocity. Oceanological and Hydrobiological Studies, 46, 199–214. 10.1515/ohs-2017-0020

[ece38013-bib-0016] Czerniawski, R., Sługocki, Ł., & Kowalska‐Góralska, M. (2016). Diurnal changes of zooplankton community reduction rate at lake outlets and related environmental factors. PLoS One, 11(7), e0158837. 10.1371/journal.pone.0158837 27392017 PMC4938256

[ece38013-bib-0017] Dorgelo, J. (1993). Growth and population structure of the zebra mussel (*Dreissena polymorpha*) in Dutch lakes differing in trophic state. In T. F. Nalepa, & D. Schloesser (Eds.), Zebra Mussels: Biology, Impacts, and Control (pp. 79–94). Lewis.

[ece38013-bib-0018] Dzierżyńska‐Białończyk, A., Jermacz, Ł., Maćkiewicz, T., Gajewska, J., & Kobak, J. (2018). Mechanisms and impact of differential fouling of the zebra mussel *Dreissena polymorpha* on different unionid bivalves. Freshwater Biology, 63, 687–699.

[ece38013-bib-0019] Gray, M. S. (2005). The role of small lake‐outlet streams in the dispersal of zebra mussel (*Dreissena polymorpha*) veligers in the upper Susquehanna River basin in New York. Occ. 41. SUNY Biol. Fld. Sta., SUNY Oneonta.

[ece38013-bib-0020] Hayes, J. W., Hughes, N. F., & Kelly, L. H. (2007). Process‐based modelling of invertebrate drift transport, net energy intake and reach carrying capacity for drift‐feeding salmonids. Ecological Modelling, 207, 171–188. 10.1016/j.ecolmodel.2007.04.032

[ece38013-bib-0021] Hieber, M., Robinson, C. T., Uehlinger, U., & Ward, J. V. (2002). Are alpine lake outlets less harsh than other alpine streams? Archiv Fur Hydrobiologie, 154, 199–223. 10.1127/archiv-hydrobiol/154/2002/199

[ece38013-bib-0022] Horvath, T. G., & Lamberti, G. A. (1997). Drifting macrophytes as a mechanism for zebra mussel (*Dreissena polymorpha*) invasion of lake‐stream outlets. The American Midland Naturalist, 138, 29–36.

[ece38013-bib-0023] Horvath, T. G., & Lamberti, G. A. (1999). Mortality of zebra mussel, *Dreissena polymorpha*, veligers during downstream transport. Freshwater Biology, 42, 69–76.

[ece38013-bib-0024] Horvath, T. G., Lamberti, G. A., Lodge, D. M., & Perry, W. L. (1996). Zebra mussel dispersal in lake‐stream systems: Source‐sink dynamics? Journal of the North American Benthological Society, 15, 564–575. 10.2307/1467807

[ece38013-bib-0025] Horvath, T., Martin, K. M., & Lamberti, G. A. (1999). Effect of Zebra Mussels, *Dreissena polymorpha*, on Macroinvertebrates in a Lake‐outlet Stream. The American Midland Naturalist, 142, 340–347.

[ece38013-bib-0026] Jasnowski, M., & Jasnowska, J. (1982). Rezerwat krajobrazowo–wodny „Rzeka Drawa” na Pomorzu Zachodnim. Chrońmy Przyrodę Ojczystą, 38(3), 5–19. [in Polish].

[ece38013-bib-0027] Jones, B. A., & McDermott, S. M. (2018). Health impacts of invasive species through an altered natural environment: Assessing air pollution sinks as a causal pathway. Environmental and Resource Economics, 71, 23–43. 10.1007/s10640-017-0135-6

[ece38013-bib-0028] Karatayev, A. Y., Boltovskoy, D., Padilla, D. K., & Burlakova, L. E. (2007). The invasive bivalves *Dreissena polymorpha* and *Limnoperna fortunei*: Parallels, contrasts, potential spread and invasion impacts. Journal of Shellfish Research, 26, 205–213.

[ece38013-bib-0029] Kobak, J., & Ryńska, A. (2014). Environmental factors affecting behavioural responses of an invasive bivalve to conspecific alarm cues. Animal Behavior, 96, 177–186. 10.1016/j.anbehav.2014.08.014

[ece38013-bib-0030] Łabęcka, A. M., & Domagala, J. (2018). Continuous reproduction of *Sinanodonta woodiana* (Lea, 1824) females: An invasive mussel species in a female‐biased population. Hydrobiologia, 810, 57–76. 10.1007/s10750-016-2835-2

[ece38013-bib-0031] Lancaster, J., Hildrew, A. G., & Gjerlov, C. (1996). Invertebrate drift and longitudinal transport processes in streams. Canadian Journal of Fisheries and Aquatic Sciences, 53, 572–582. 10.1139/f95-217

[ece38013-bib-0032] Lazareva, V. I., Kopylov, A. I., Sokolova, E. A., & Pryanichnikova, E. G. (2016). Veliger larvae of dreissenids (Bivalvia, Dreissenidae) in the plankton foodweb of Rybinsk Reservoir. Biology Bull., 43, 1313–1321. 10.1134/S1062359016100083

[ece38013-bib-0033] Leuven, R. S., van der Velde, G., Baijens, I., Snijders, J., van der Zwart, C., Lenders, H. R., & bij de Vaate, A. (2009). The river Rhine: A global highway for dispersal of aquatic invasive species. Biological Invasions, 11, 1989. 10.1007/s10530-009-9491-7

[ece38013-bib-0034] Martin, M. D., Mackie, G. L., & Baker, M. A. (1993). Control of the biofouling mollusc, *Dreissena polymorpha* (Bivalvia: Dreissenidae), with sodium hypochlorite and with polyquaternary ammonia and benzothiazole compounds. Archives of Environmental Contamination and Toxicology, 24, 381–388. 10.1007/BF01128738

[ece38013-bib-0035] Mehler, K., Burlakova, L. E., Karatayev, A. Y., Biesinger, Z., Valle‐Levinson, A., Castiglione, C., & Gorsky, D. (2018). Sonar technology and underwater imagery analysis can enhance invasive *Dreissena* distribution assessment in large rivers. Hydrobiologia, 810, 119–131. 10.1007/s10750-016-3040-z

[ece38013-bib-0036] Miller, S. J., & Haynes, J. M. (1997). Factors limiting colonization of western New York creeks by the zebra mussel (*Dreissena polymorpha*). Journal of Freshwater Ecology, 12, 81–88.

[ece38013-bib-0037] Mills, E. L., Adams, C., O'Gorman, R., Owens, R. W., & Roseman, E. F. (1995). Planktivory by alewife (*Alosa pseudoharengus*) and rainbow smelt (*Osmerus mordax*) on microcrustacean zooplankton and dreissenid (Bivalvia: Dreissenidae) veligers in southern Lake Ontario. Canadian Journal of Fisheries and Aquatic Sciences, 52, 925–935.

[ece38013-bib-0038] Mills, E. L., Dermott, R. M., Roseman, E. F., Dustin, D., Mellina, E., Conn, D. B., & Spidle, A. P. (1993). Colonization, ecology, and population structure of the "quagga'' mussel (Bivalvia: Dreissenidae) in the lower Great Lakes. Canadian Journal of Fisheries and Aquatic Sciences, 50, 2305–2314. 10.1139/f93-255

[ece38013-bib-0039] Mills, E. L., Rosenberg, G., Spidle, A. P., Ludyanskiy, M., Pligin, Y., & May, B. (1996). A review of the biology and ecology of the quagga mussel (*Dreissena bugensis*), a second species of freshwater dreissenid introduced to North America. American Zoologist, 36, 271–286.

[ece38013-bib-0040] Molloy, D. P., Mayer, D. A., Gaylo, M. J., Morse, J. T., Presti, K. T., Sawyko, P. M., Karatayev, A. Y., Burlakova, L. E., Laruelle, F., Nishikawa, K. C., & Griffin, B. H. (2013). Pseudomonas fluorescens strain CL145A–A biopesticide for the control of zebra and quagga mussels (Bivalvia: Dreissenidae). Journal of Invertebrate Pathology, 113, 104–114. 10.1016/j.jip.2012.12.012 23295683

[ece38013-bib-0041] Mouton, T. L., Matheson, F. E., Stephenson, F., Champion, P. D., Wadhwa, S., Hamer, M. P., Catlin, A., & Riis, T. (2019). Environmental filtering of native and non‐native stream macrophyte assemblages by habitat disturbances in an agricultural landscape. Science of the Total Environment, 659, 1370–1381. 10.1016/j.scitotenv.2018.12.277 31096347

[ece38013-bib-0042] Oksanen, J., Blanche, F. G., Friendly, M., Kindt, R., Legendre, P., & McGlinn, D. (2020). Vegan: Community Ecology Package. R package version 2.5‐7. https://CRAN.R‐project.org/package=vegan

[ece38013-bib-0043] Orlova, M., Golubkov, S., Kalinina, L., & Ignatieva, N. (2004). *Dreissena polymorpha* (Bivalvia: Dreissenidae) in the Neva Estuary (eastern Gulf of Finland, Baltic Sea): Is it a biofilter or source for pollution? Marine Pollution Bulletin, 49, 196–205. 10.1016/j.marpolbul.2004.02.008 15245984

[ece38013-bib-0044] Pace, M. L., Findlay, S. E., & Fischer, D. (1998). Effects of an invasive bivalve on the zooplankton community of the Hudson River. Freshwater Biology, 39, 103–116. 10.1046/j.1365-2427.1998.00266.x

[ece38013-bib-0045] Palmer, M. A., Menninger, H. L., & Bernhardt, E. (2010). River restoration, habitat heterogeneity and biodiversity: A failure of theory or practice? Freshwater Biology, 55, 205–222. 10.1111/j.1365-2427.2009.02372.x

[ece38013-bib-0046] Patterson, M. W., Ciborowski, J. J., & Barton, D. R. (2005). The distribution and abundance of *Dreissena* species (Dreissenidae) in Lake Erie, 2002. Journal of Great Lakes Research, 31, 223–237. 10.1016/S0380-1330(05)70316-6

[ece38013-bib-0047] Pourriot, R., Rougier, C., & Miquelis, A. (1997). Origin and development of river zooplankton: Example of the Marne. Hydrobiologia, 345, 143–148.

[ece38013-bib-0048] R Core Team (2020). R: A language and environment for statistical computing. Vienna, Austria: R Foundation for Statistical Computing. https://www.R‐project.org/

[ece38013-bib-0049] Rehman, C. R., Stoeckel, J. A., & Schneider, D. W. (2003). Effect of turbulence on the mortality of zebra mussel veligers. Canadian Journal of Zoology, 81, 1063–1069. 10.1139/z03-090

[ece38013-bib-0050] Ricciardi, A., Serrouya, R., & Whoriskey, F. G. (1995). Aerial exposure tolerance off zebra and quagga mussels (Bivalvia: Dreissenidae): Implications for overland dispersal. Canadian Journal of Fisheries and Aquatic Sciences, 52, 470–477. 10.1139/f95-048

[ece38013-bib-0051] Richardson, J. S., & Mackay, R. J. (1991). Lake outlets and the distribution of filter feeders: An assessment of hypotheses. Oikos, 62, 370–380. 10.2307/3545503

[ece38013-bib-0052] Sanz‐Ronda, F. J., Lopez‐Saenz, S., San‐Martın, R., & Palau‐Ibars, A. (2014). Physical habitat of zebra mussel (*Dreissena polymorpha*) in the lower Ebro River (Northeastern Spain): Influence of hydraulic parameters in their distribution. Hydrobiologia, 735, 137–147. 10.1007/s10750-013-1638-y

[ece38013-bib-0053] Schulz, M., Kozerski, H. P., Pluntke, T., & Rinke, K. (2003). The influence of macrophytes on sedimentation and nutrient retention in the lower River Spree (Germany). Water Research, 37, 569–578. 10.1016/S0043-1354(02)00276-2 12688691

[ece38013-bib-0054] Simberloff, D. (2014). Biological invasions: What's worth fighting and what can be won? Ecological Engineering, 65, 112–121. 10.1016/j.ecoleng.2013.08.004

[ece38013-bib-0055] Sousa, R., Novais, A., Costa, R., & Strayer, D. L. (2014). Invasive bivalves in fresh waters: Impacts from individuals to ecosystems and possible control strategies. Hydrobiologia, 735, 233–251. 10.1007/s10750-012-1409-1

[ece38013-bib-0056] Sousa, R., Pilotto, F., & Aldridge, D. C. (2011). Fouling of European freshwater bivalves (Unionidae) by the invasive zebra mussel (*Dreissena polymorpha*). Freshwater Biology, 56, 867–876. 10.1111/j.1365-2427.2010.02532.x

[ece38013-bib-0057] Stańczykowska, A., & Lewandowski, K. (1992). Thirty years of studies of *Dreissena polymorpha* ecology in Mazurian lakes in Northern Poland. In T. Nalepa, & D. W. Schloesser (Eds.), Zebra mussels biology, impacts, and control (pp. 3–37). CRC Press.

[ece38013-bib-0058] Stępień, E., Zawal, A., Buczyński, P., Buczyńska, E., & Szenejko, M. (2019). Effects of dredging on the vegetation in a small lowland river. PeerJ, 7, e6282. 10.7717/peerj.6282 30697485 PMC6346983

[ece38013-bib-0059] Szyper, H., & Gołdyn, R. (2002). Role of catchment area in the transport of nutrients to lakes in the Wielkopolska National Park in Poland. Lake and Reservoir Management, 7, 25–33. 10.1046/j.1440-1770.2002.00164.x

[ece38013-bib-0060] Taeubert, J. E., El‐Nobi, G., & Geist, J. (2014). Effects of water temperature on the larval parasitic stage of the thick‐shelled river mussel (*Unio crassus*). Aquatic Conservation: Marine and Freshwater Ecosystems, 24, 231–237.

[ece38013-bib-0061] Wacker, A., & Elert, E. (2003). Food quality controls reproduction of the zebra mussel (*Dreissena polymorpha*). Oecologia, 135, 332–338. 12721821 10.1007/s00442-003-1208-5

[ece38013-bib-0062] Winkel, E. H. T., & Davids, C. (1982). Food selection by *Dreissena polymorpha* Pallas (Mollusca: Bivalvia). Freshwater Biology, 12, 553–558. 10.1111/j.1365-2427.1982.tb00647.x

[ece38013-bib-0063] Wohl, E., Lane, S. N., & Wilcox, A. C. (2015). The science and practice of river restoration. Water Resources Research, 51, 5974–5997. 10.1002/2014WR016874

[ece38013-bib-0064] Wojtal‐Frankiewicz, A., & Frankiewicz, P. (2011). The impact of pelagic (*Daphnia longispina*) and benthic (*Dreissena polymorpha*) filter feeders on chlorophyll and nutrient concentration. Limnologica, 41, 191–200. 10.1016/j.limno.2010.09.001

[ece38013-bib-0065] Wojtal‐Frankiewicz, A., Sieczko, A., Izydorczyk, K., Jurczak, T., & Frankiewicz, P. (2010). Competitive influence of Zebra Mussel (*Dreissena polymorpha*) on *Daphnia longispina* population dynamics in the presence of cyanobacteria. International Review of Hydrobiology, 95, 313–329. 10.1002/iroh.201011241

[ece38013-bib-0066] Wong, W. H., & Levinton, J. (2005). Consumption rates of two rotifer species by zebra mussels *Dreissena polymorpha* . Marine and Freshwater Behaviour and Physiology, 38, 149–159. 10.1080/13638490500174699

[ece38013-bib-0067] Wotton, R. S. (1995). Temperature and lake‐outlet communities. Journal of Thermal Biology, 1, 121–125. 10.1016/0306-4565(94)00042-H

[ece38013-bib-0068] Zerebecki, R. A., & Sorte, C. J. (2011). Temperature tolerance and stress proteins as mechanisms of invasive species success. PLoS One, 6(4), e14806. 10.1371/journal.pone.0014806 21541309 PMC3082523

