# Supplementary material for: Does lake eutrophication support biological invasions in rivers? A study on Dreissena polymorpha (Bivalvia) in lake–river ecotones
Source: Ecol Evol. 2021 Aug 13;11(18):12686–96. doi: 10.1002/ece3.8013 (PMC8462168; doi:10.1002/ece3.8013)
Supplement: Supplementary file 1 — Table S1 [file ECE3-11-12686-s001.docx]

**Table S 1.** Abundance od bivalves (Dreissenidae, Sphaeridae, Unionidae) (ind. m^-2^) and values of environmental conditions at examined outlets.

| Outlet number | Dreissenidae | Sphaeriidae | Unionidae | Carlson index | Depth | Width | Current velocity | Vegetation coverage | Bottom type |
| --- | --- | --- | --- | --- | --- | --- | --- | --- | --- |
|  | (ind. m^-2^) | (ind. m^-2^) | (ind. m^-2^) |  | (m) | (m) | (m s^-1^) | (%) |  |
| 1 | 0 | 32 | 4 | 52 | 0.1 | 0.15 | 0.02 | 10 | POM/sand |
| 2 | 0 | 48 | 0 | 54 | 0.1 | 0.2 | 0.02 | 100 | POM/sand |
| 3 | 0 | 752 | 0 | 57 | 0.1 | 0.5 | 0.02 | 0 | POM/sand |
| 4 | 0 | 4 | 0 | 58 | 0.2 | 0.7 | 0.04 | 10 | POM/sand |
| 5 | 0 | 952 | 0 | 60 | 0.2 | 1.5 | 0.05 | 80 | POM/sand |
| 6 | 0 | 16 | 0 | 54 | 0.2 | 4 | 0.1 | 20 | POM/sand |
| 7 | 12 | 48 | 8 | 58 | 0.3 | 4 | 0.35 | 0 | sand/stones |
| 8 | 232 | 2336 | 0 | 52 | 0.2 | 3 | 0.28 | 20 | POM/sand |
| 9 | 3884 | 24 | 8 | 55 | 0.5 | 7 | 0.38 | 0 | sand/gravel |
| 10 | 2148 | 80 | 8 | 58 | 0.4 | 8 | 0.37 | 5 | sand/gravel |
| 11 | 0 | 8 | 0 | 58 | 0.15 | 3 | 0.1 | 80 | POM/sand |
| 12 | 0 | 32 | 0 | 48 | 0.1 | 0.7 | 0.1 | 0 | sand |
| 13 | 0 | 112 | 0 | 39 | 0.2 | 3 | 0.2 | 10 | sand |
| 14 | 0 | 864 | 0 | 48 | 0.2 | 1 | 0.03 | 80 | POM/sand |
| 15 | 44 | 72 | 32 | 51 | 0.2 | 3 | 0.3 | 60 | sand/gravel |
| 16 | 0 | 280 | 0 | 47 | 0.1 | 0.7 | 0.2 | 60 | POM/sand |
| 17 | 0 | 32 | 0 | 47 | 0.1 | 0.4 | 0.08 | 60 | POM/sand |
| 18 | 0 | 616 | 16 | 57 | 0.2 | 2 | 0.18 | 1 | sand |
| 19 | 160 | 160 | 0 | 58 | 0.4 | 20 | 0.4 | 10 | sand/stones |
| 20 | 0 | 8 | 0 | 57 | 0.1 | 0.7 | 0.05 | 80 | POM/sand |
| 21 | 0 | 60 | 12 | 63 | 0.9 | 22 | 0.11 | 20 | POM/sand |
| 22 | 0 | 264 | 0 | 53 | 0.2 | 1 | 0.16 | 20 | sand |
| 23 | 40 | 84 | 4 | 58 | 1.2 | 22 | 0.23 | 30 | sand/gravel |
| 24 | 0 | 392 | 20 | 57 | 0.2 | 5 | 0.2 | 60 | POM/sand |
| 25 | 0 | 40 | 0 | 44 | 0.25 | 5 | 0.15 | 10 | POM/sand |
| 26 | 34 | 28 | 0 | 51 | 0.7 | 14 | 0.21 | 40 | sand/gravel |
| 27 | 0 | 16 | 0 | 63 | 0.2 | 2 | 0.2 | 30 | POM/sand |
| 28 | 7 | 124 | 0 | 63 | 0.3 | 5 | 0.21 | 5 | sand |
| 29 | 0 | 310 | 0 | 62 | 0.2 | 1.5 | 0.07 | 30 | sand |
| 30 | 0 | 16 | 0 | 40 | 0.1 | 1.6 | 0.06 | 80 | POM/sand |
| 31 | 14 | 20 | 4 | 63 | 0.2 | 2.5 | 0.22 | 10 | POM/sand |
| 32 | 24 | 16 | 0 | 59 | 0.9 | 16 | 0.39 | 10 | sand/gravel |
| 33 | 40 | 2416 | 0 | 48 | 0.2 | 1.5 | 0.24 | 10 | sand |
| 34 | 0 | 88 | 0 | 40 | 0.2 | 6 | 0.1 | 0 | sand |
| 35 | 0 | 432 | 0 | 46 | 0.2 | 3 | 0.2 | 0 | sand |
| 36 | 0 | 20 | 0 | 60 | 0.3 | 7 | 0.28 | 50 | POM/sand |
| 37 | 41 | 16 | 0 | 65 | 0.15 | 2 | 0.03 | 30 | sand/gravel |
| 38 | 0 | 48 | 0 | 37 | 0.1 | 0.7 | 0.05 | 80 | POM/sand |
| 39 | 0 | 4 | 0 | 53 | 0.1 | 1.2 | 0.03 | 80 | POM/sand |
| 40 | 0 | 24 | 0 | 56 | 0.1 | 0.3 | 0.02 | 80 | POM/sand |
| 41 | 0 | 244 | 0 | 53 | 0.05 | 0.3 | 0.01 | 80 | POM/sand |
| 42 | 0 | 536 | 0 | 63 | 0.1 | 2 | 0.07 | 20 | POM/sand |
| 43 | 0 | 32 | 0 | 42 | 0.1 | 2 | 0.03 | 50 | POM/sand |
| 44 | 0 | 8 | 16 | 57 | 0.2 | 2.5 | 0.1 | 60 | POM/sand |
| 45 | 8 | 376 | 0 | 47 | 0.3 | 4 | 0.2 | 0 | sand/gravel |
| 46 | 10 | 15 | 0 | 65 | 0.3 | 6 | 0.4 | 0 | sand/stones |
| 47 | 0 | 4 | 0 | 47 | 0.5 | 8 | 0.6 | 10 | sand/gravel |
| 48 | 0 | 4 | 0 | 38 | 0.2 | 10 | 0.5 | 1 | sand/gravel |
| 49 | 12 | 40 | 0 | 65 | 0.5 | 6 | 0.3 | 10 | sand/gravel |
| 50 | 0 | 120 | 32 | 49 | 0.5 | 8 | 0.3 | 30 | POM/sand |
| 51 | 0 | 144 | 8 | 54 | 0.4 | 11 | 0.5 | 10 | sand/gravel |
| 52 | 3456 | 224 | 96 | 58 | 0.3 | 4 | 0.21 | 50 | POM/sand |
| 53 | 0 | 1080 | 0 | 51 | 0.05 | 0.7 | 0.06 | 2 | POM/sand |
| 54 | 0 | 24 | 0 | 49 | 0.1 | 1 | 0.04 | 70 | sand |
| 55 | 0 | 520 | 0 | 63 | 0.1 | 3 | 0.18 | 60 | POM/sand |
